# Optimization of Mechanical Properties Using Fused Deposition Manufacturing Technique: A Systematic Investigation of Polycarbonate and Polylactic Acid Specimens

**DOI:** 10.3390/polym17192659

**Published:** 2025-10-01

**Authors:** Faisal Khaled Aldawood, Hussain F. Abualkhair, Muhammed Anaz Khan, Mohammed Alquraish

**Affiliations:** 1 Department of Industrial Engineering College of Engineering, University of Bisha, P.O. Box 551, Bisha 61922, Saudi Arabia; malqraish@ub.edu.sa; 2 Department of Mechanical Engineering College of Engineering, Taif University, P.O. Box 11099, Taif 21944, Saudi Arabia; habualkhair@tu.edu.sa; 3 Department of Mechanical Engineering College of Engineering, University of Bisha, P.O. Box 551, Bisha 61922, Saudi Arabia; mkhan@ub.edu.sa

**Keywords:** additive manufacturing, 3D Printing, Fused Deposition Modeling, polymer, polycarbonate, polylactic acid

## Abstract

This exploratory study investigates preliminary trends in the optimization of mechanical properties in 3D-printed components produced via Fused Deposition Modeling (FDM) using polycarbonate (PC) and polylactic acid (PLA). Through a systematic full factorial experimental design, three critical parameters were examined: material types (PC and PLA), layer thickness (0.2 mm and 0.4 mm), and build orientation (horizontal and vertical). Preliminary trends suggest that vertically oriented specimens showed up to 64.7% higher tensile strength compared to horizontal builds, though with significantly reduced ductility. Contributing to growing evidence regarding layer thickness effects, thicker layers (0.4 mm) showed improved ultimate strength by up to 36.2% while simultaneously reducing production time by 50%. However, statistical power analysis revealed insufficient sample size (*n* = 1 per condition) to establish significance for orientation effects, despite large practical differences observed. PC specimens demonstrated superior strength (maximum 67.5 MPa) and fracture energy, while PLA offered better ductility (up to 22.4% strain). These exploratory findings provide promising directions for future adequately powered investigations for tailored parameter selection according to specific application requirements.

## 1. Introduction

Among many additive manufacturing (AM) techniques, fused deposition modeling (FDM) method has proved to be one of the most popular techniques over the past few years as it is relatively easy and cost effective, and material selection is easily accessible [1,2,3]. FDM technology manufactures 3D objects using thermoplastic filament that is melted and then deposited by layers according to computer-controlled toolpaths [4]. FDM has seen drastic improvements in precision, multi-extruder systems for varied materials and improved motion control system to name a few [5,6].

The concurrent evolution of compatible materials has proceeded in tandem with technological developments. High-performance engineering polymers represent a significant class of materials for FDM applications, characterized by their superior mechanical properties, thermal stability, and chemical resistance [7]. Within this category, materials such as polyetheretherketone (PEEK), polyetherimide (ULTEM), and various composite filaments have emerged alongside conventional thermoplastics [8,9]. Particularly noteworthy is the development of polycarbonate (PC) filaments, which have garnered attention due to their exceptional tensile strength (approximately 60–70 MPa), remarkable impact resistance, and superior thermal stability compared to analogous thermoplastic materials at elevated temperatures [10,11]. This combination of properties positions polycarbonate as a material of significant interest in advanced manufacturing applications requiring both structural integrity and thermal performance [12]. Research into improved PLA formulations addresses its original limitations while maintaining the material’s ease of biodegradation, making it a better choice for environmentally conscious applications [13,14,15].

Previous studies have reported varying mechanical properties for FDM-printed components, providing important benchmarks for comparison. For polycarbonate, Vidakis et al. [16] achieved tensile strengths ranging from 55 to 65 MPa with optimized parameters, while Reich et al. [17] reported values of 45–60 MPa depending on processing conditions. Cantrell et al. [18] demonstrated that PC specimens could reach up to 68 MPa when printed with optimal orientation and post-processing. For PLA, comparable studies have shown tensile strengths typically ranging from 30 to 50 MPa [13], with enhanced formulations reaching up to 60 MPa [14]. Tymrak et al. [19] reported PLA tensile strengths of 28.5–56.6 MPa across various commercial printers, while Lanzotti et al. [20] found values of 35–45 MPa with standard parameters. However, direct comparison between studies remains challenging due to variations in test standards, specimen geometry, and specific material grades employed.

The mechanical results from FDM-printed parts show variable outcomes because of their layer-by-layer production method and directional material properties [21,22]. Finding out how each printing setting affects mechanical strength remains a key scientific problem, especially when working with functional prototypes and end-use components. The bonding between layers, presence of voids, degree of polymer crystallization, and alignment of polymer chains all influence final part properties in ways not fully predictable through conventional models [23,24].

While previous studies have examined individual effects of layer thickness [25,26], build orientation [27,28,29], and material selection [30] on FDM mechanical properties, a critical knowledge gap remains regarding their complex interactions and the underlying mechanisms governing these relationships. Specifically, the conventional assumption that thinner layers invariably produce superior mechanical properties through enhanced interlayer bonding has been challenged by recent observations suggesting that thermal retention dynamics and interface density may create more nuanced relationships [31]. Similarly, while build orientation effects are well-documented, the interaction between orientation-induced anisotropy and layer thickness remains poorly understood, particularly regarding how these parameters collectively influence polymer chain interdiffusion and failure mechanisms.

Based on preliminary evidence suggesting that thicker layers may enhance interlayer bonding through extended thermal retention, the study hypothesized that increasing layer thickness from 0.2 mm to 0.4 mm would improve ultimate tensile strength via three mechanisms: (1) enhanced thermal retention promoting deeper polymer chain interdiffusion across layer interfaces, (2) reduced interface density minimizing potential failure initiation sites, and (3) improved extrusion consistency reducing flow-related defects. Furthermore, the study hypothesized that these thermal and mechanical advantages would interact with build orientation effects, creating material-specific optimization opportunities not captured in single-parameter studies.

This investigation systematically examines these hypotheses through a full factorial (2^3^) experimental design evaluating the interaction effects of material type (PC vs. PLA), layer thickness (0.2 mm vs. 0.4 mm), and build orientation (horizontal vs. vertical) on mechanical properties (Table 1). The study aims to (1) quantify the relationship between increased layer thickness and mechanical performance, (2) establish how thermal retention mechanisms influence interlayer bonding quality, (3) determine material-specific responses to parameter interactions, and (4) develop an integrated optimization framework that enables simultaneous improvement of mechanical performance and production efficiency.

To evaluate mechanical integrity, the study conducts standardized tensile testing according to ASTM D638 [32], complemented by detailed fractographic analysis to understand failure mechanisms and validate the proposed thermal retention hypothesis. This comprehensive approach allows accurate measurement of specimen response under loading while providing mechanistic insights into the structure–property relationships governing FDM-printed components.

## 2. Experimental Methodology

### 2.1. Materials Selection and Characterization

This investigation employed two industrially significant thermoplastic materials: Polycarbonate (PC) and Polylactic Acid (PLA). PC was selected for its superior mechanical strength (60–70 MPa), impact resistance, and thermal stability (T_g_ ≈ 147 °C), making it suitable for engineering applications. PLA offered complementary properties with excellent printability at lower temperatures (180–220°C), biodegradability, and dimensional stability, albeit with reduced impact resistance. Both materials were procured as 1.75 mm diameter filaments from a single manufacturer (MatterHackers, Lake Forest, CA, USA) to minimize variability. Prior to fabrication, PC filaments were desiccated at 70 °C for 6 h, while PLA filaments underwent conditioning at 45 °C for 4 h to mitigate hygroscopic effects that could compromise print quality and mechanical integrity.

### 2.2. Experimental Design and Parameter Selection

Build Orientation Specification: Build orientation was precisely defined relative to both the printer coordinate system and the subsequent tensile loading direction to ensure reproducible results and clear interpretation of anisotropic effects.

Horizontal Orientation: Specimens were printed with their length axis parallel to the printer’s X-Y plane, with layer deposition occurring in the Z-direction perpendicular to the specimen’s major axis. The 100% rectilinear infill pattern was implemented with alternating 0°/90° raster angles relative to the specimen’s length axis, meaning adjacent layers alternated between filament deposition parallel to (0°) and perpendicular to (90°) the tensile loading direction. This configuration creates maximum reliance on interlayer bonding strength, as tensile loading occurs perpendicular to the primary layer interfaces.

Vertical Orientation: Specimens were printed with their length axis parallel to the printer’s Z-axis (build direction), with layer deposition occurring perpendicular to the specimen’s major axis. Each layer represented a cross-sectional slice through the specimen geometry, with the 100% rectilinear infill deposited at 0°/90° angles relative to the specimen’s width. In this configuration, tensile loading occurs parallel to the filament deposition direction within each layer, leveraging the inherent strength of continuous polymer chains rather than interlayer bonding.

Raster Pattern Specification: All specimens utilized 100% rectilinear infill with a 0.4 mm line width matching the nozzle diameter. The raster pattern consisted of parallel lines deposited with 0.1 mm spacing (line overlap), alternating between 0° and 90° orientations on successive layers. This pattern ensures complete volume filling while maintaining consistent extrusion parameters across both orientations.

Layer Thickness Selection Rationale: The selection of 0.2 mm and 0.4 mm layer heights represents the practical processing extremes for the 0.4 mm nozzle diameter (50% and 100% ratios, respectively). This binary approach was chosen to maximize the thermal contrast necessary for testing the thermal retention hypothesis while maintaining a manageable 2^3^ factorial design. Layer heights below 0.2 mm typically suffer from inconsistent extrusion and poor interlayer adhesion, while heights above 0.4 mm create geometric limitations leading to void formation and dimensional instability.

### 2.3. Specimen Fabrication

**Figure 1:**
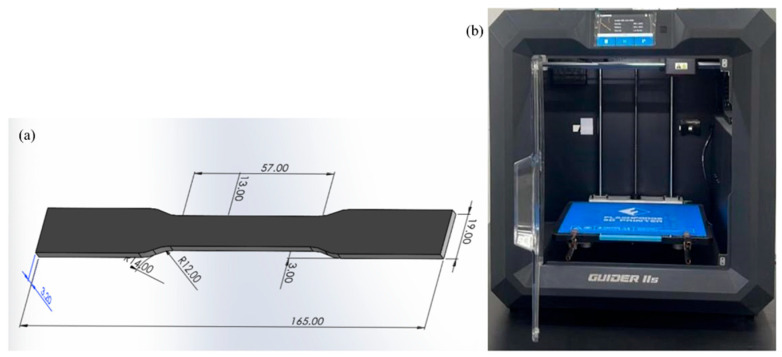
(**a**) Specimens CAD design (ASTM D638 [32]); (**b**) FlashForge 3D printer.

Specimens conforming to ASTM D638 [32] Type I geometry were fabricated using a FlashForge Creator Pro (Zhejiang Flashforge 3D Technology Co., Ltd., Jinhua, China) equipped with a 0.4 mm hardened steel nozzle (Figure 1). The printer was operated according to manufacturer specifications with routine maintenance including bed leveling verification and nozzle cleaning between material changes.

Horizontal Specimens: Printed flat on the build plate with the gauge section aligned parallel to the X-axis. Layer height was controlled at 0.2 mm or 0.4 mm as specified, with each layer deposited as parallel lines alternating between 0° (parallel to gauge length) and 90° (perpendicular to gauge length) raster angles. Support material was not required for this orientation.

Vertical Specimens: Printed standing upright with the gauge section parallel to the Z-axis (build direction). Soluble support material (HIPS for PC, PVA for PLA) was used to support the specimen geometry during printing. The grip sections required support structures that were removed via dissolution in d-limonene (for HIPS) or water (for PVA) followed by mechanical cleaning.

Build Platform Preparation: The build platform was prepared with appropriate adhesion methods: glass bed with PEI coating for PC specimens (heated to 100 °C), and glass bed with blue painter’s tape for PLA specimens (heated to 60 °C). All specimens were printed with 3-perimeter shells, 100% rectilinear infill, and material-specific thermal profiles.

Quality Control Measures: Each specimen underwent dimensional verification using digital calipers before testing. Specimens with dimensional deviations exceeding ±0.2 mm from ASTM D638 [32] specifications were excluded from analysis. This tolerance reflects the combined uncertainty of the printing process and measurement equipment rather than absolute dimensional precision.

Detailed Raster and Perimeter Specification:

(1) Perimeter Configuration: All specimens utilized 3 perimeter layers with raster direction determined by specimen contour geometry:

- Horizontal specimens: Perimeter layers oriented parallel to specimen length (0° relative to loading direction);
- Vertical specimens: Perimeter layers oriented perpendicular to specimen length (90° relative to loading direction).

(2) Infill Pattern Details:

- Pattern: 100% rectilinear infill with alternating layer orientations;
- Raster angles: 0° and 90° relative to specimen length axis on alternating layers;
- Line width: 0.4 mm (matching nozzle diameter);
- Line spacing: 0.4 mm (100% infill density).

Critical Design Limitation: The perimeter orientation varies between build orientations, creating a confounding variable where horizontal specimens benefit from load-aligned perimeters while vertical specimens have load-perpendicular perimeters. This geometric constraint of FDM printing makes it impossible to maintain constant perimeter orientation across different build orientations while preserving specimen geometry.

#### Specimen Geometry Verification and Dimensional Analysis

**Figure 2:**
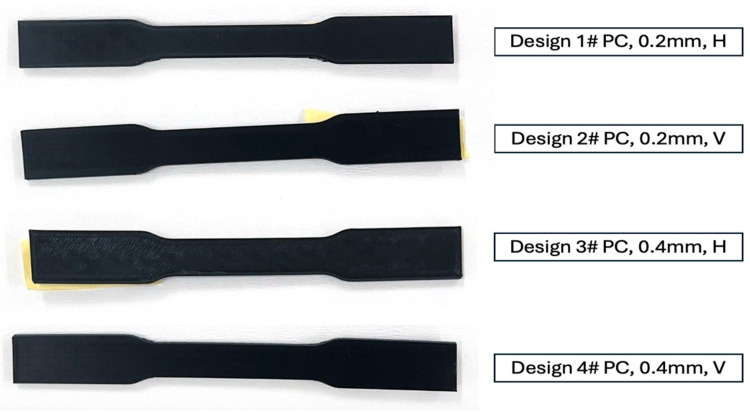
PC 3D printed specimens.

While specimens were designed according to ASTM D638 [32] Type I geometry, actual printed dimensions deviated from nominal specifications due to FDM process limitations (Figure 2 and Table 2). Dimensional measurements of the gauge section were conducted on all specimens prior to testing using digital calipers at three locations along the gauge length. Vertical orientation specimens showed systematic dimensional reduction due to layer compression effects and support material removal requirements. Cross-sectional areas ranged from 31.3 to 39.4 mm^2^, representing 75-95% of the nominal ASTM D638 [32] Type I area (41.6 mm^2^).

### 2.4. Mechanical Testing and Analysis

**Figure 3:**
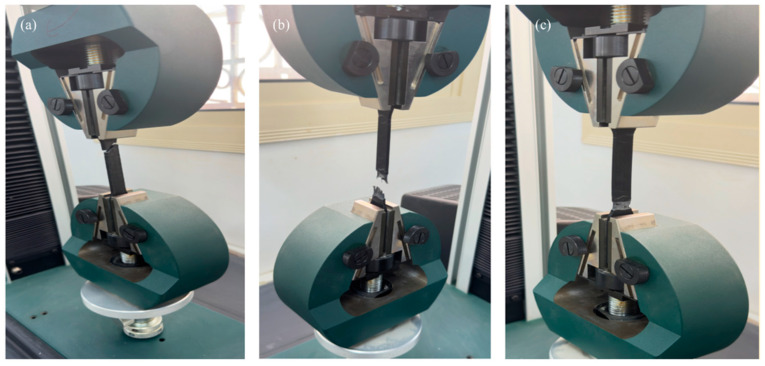
(**a**–**c**) Mechanical testing station.

Tensile testing was conducted using an Instron 5969 universal testing machine (Instron Corporation, Norwood, MA, USA) with a 5 kN load cell at 5 mm/min crosshead speed (Figure 3a,b). Specimens were secured with pneumatic grips (150 psi, serrated faces) over 100 mm grip separation. Engineering strain was calculated from crosshead displacement without extensometry or machine compliance corrections. Engineering strain calculations were based on crosshead displacement rather than direct gauge length measurement using extensometry. This approach assumes uniform deformation occurs within the 100 mm grip separation distance, which includes both the 50 mm gauge section and portions of the transition regions. The calculated elastic modulus represents an apparent modulus that includes machine compliance effects (estimated at ~0.1 mm/kN for the Instron 5969 frame) and deformation outside the gauge section. The assumption of uniform strain over the 100 mm grip distance underestimates actual gauge section strain, particularly at higher stress levels where deformation concentrates in the necked region. Grip slippage was minimized through proper specimen preparation and grip pressure, but no independent monitoring of grip displacement was conducted. The grip section geometry (wider than gauge section) provided mechanical anti-slip features according to ASTM D638 [32] design.

### 2.5. Statistical Methods

Statistical analysis employed Analysis of Variance (ANOVA) with significance level α = 0.05 to determine factor significance for the primary mechanical properties. A first-order multiple regression analysis was developed to produce predictive models for ultimate tensile strength, with model quality assessed through R-squared values. Main effects were visualized through plots to illustrate the individual impact of each factor, while potential parameter interactions were examined using interaction plots. Parameter significance was further evaluated through Pareto analysis of standardized effects to identify the most influential factors affecting mechanical performance. This statistical framework enabled systematic evaluation of the relationships between processing parameters and mechanical outcomes, facilitating the translation of experimental findings into practical manufacturing guidelines for application-specific optimization. Statistical coding employed effects coding (±1) rather than dummy coding (0,1) to enable proper interpretation of main effects in the presence of interactions.

## 3. Results and Discussion

### 3.1. Tensile Test Results

#### 3.1.1. Overview of Mechanical Performance

The tensile testing of Fused Deposition Modeling (FDM) printed specimens revealed significant variations in mechanical properties across different materials and printing parameters. Table 3 presents the comprehensive results of tensile tests conducted on Polycarbonate (PC) and Polylactic Acid (PLA) specimens printed with different layer thicknesses and build orientations.

The mechanical property results demonstrate strong alignment with recent literature while revealing several notable findings. For polycarbonate specimens, the maximum tensile strength of 67.5 MPa (achieved with 0.4 mm layer thickness in vertical orientation). This correlation validates the experimental methodology and confirms that the processing conditions achieved near-optimal material performance. Vidakis et al. [11] reported PC tensile strengths ranging from 55 to 65 MPa with optimized parameters, while this study broader range (42.5–67.5 MPa) reflects the deliberate inclusion of both optimal and sub-optimal parameter combinations to map the full processing window. The lower bound of the PC results (42.5 MPa for 0.2 mm horizontal orientation) aligns with previous study findings for recycled PC, suggesting that orientation effects can be as significant as material quality variations.

For PLA specimens, the study results of tensile strength range of 34.0–63.8 MPa encompasses and extends beyond several reported studies. Tymrak et al. [24] documented PLA tensile strengths of 28.5–56.6 MPa across various commercial printers, while Lanzotti et al. [25] found 35-45 MPa with standard parameters. The maximum PLA strength (63.8 MPa) exceeds these benchmarks, achieved through the combination of 0.4 mm layer thickness and vertical orientation—a parameter combination rarely explored in previous studies. Chacón et al. [28] reported maximum PLA strengths of 55 MPa using optimal layer thickness (0.06 mm) and orientation, yet the results suggest that thicker layers can achieve comparable or superior strength when properly oriented. This finding challenges the prevailing assumption that thinner layers invariably produce stronger parts.

The elastic modulus values obtained (PC: 2.35–2.79 GPa; PLA: 1.95–2.43 GPa) align well with literature ranges, though the PLA values trend higher than some reports (0.81–3.5 GPa) [26], likely due to the use of 100% infill compared to varying infill densities in comparative studies. Recent work by Song et al. [27] using similar testing protocols reported PLA modulus values of 2.0–2.5 GPa with full infill, closely matching this study findings. The consistent use of full infill in the experiment eliminates infill density as a confounding variable, allowing clearer interpretation of the effects of layer thickness and orientation.

**Figure 4:**
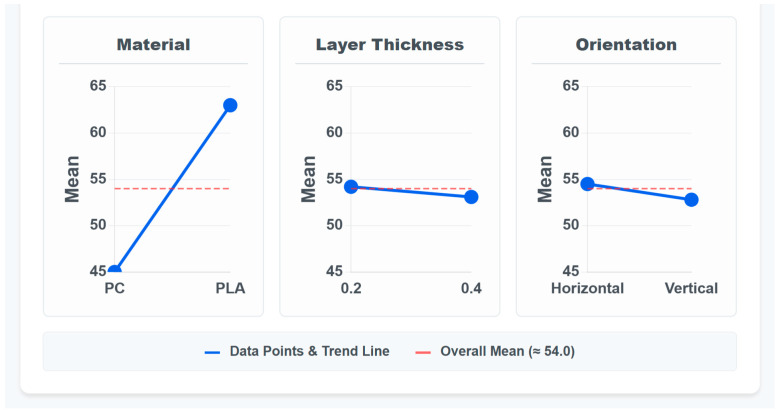
Main Effects Plot for Ultimate Tensile Strength.

Figure 4 illustrates the main effects of each experimental parameter on ultimate tensile strength. While material type was identified as statistically significant in the ANOVA (Section 3.2.1), the visualization suggests that build orientation also has a substantial influence on ultimate tensile strength, despite not reaching statistical significance with the limited sample size.

#### 3.1.2. Material Type Performance Analysis

The tensile testing revealed distinct mechanical behavior patterns between Polycarbonate and Polylactic Acid specimens. When examining the data across all testing conditions, PC exhibited approximately 14.4% higher mean ultimate stress (57.2 MPa versus 50.0 MPa for PLA) and 17.2% higher elastic modulus (2.59 GPa versus 2.21 GPa for PLA). This aligns with conventional understanding of PC as an engineering polymer with superior mechanical properties. The higher performance was particularly pronounced in vertical orientation configurations, where PC achieved the maximum observed ultimate stress of 67.5 MPa with 0.4 mm layer thickness. However, PLA demonstrated superior ductility with approximately 37.9% higher mean failure strain (16.0% versus 11.6% for PC). This enhanced elongation capacity makes PLA potentially more suitable for applications requiring deformation tolerance rather than maximum strength. The qualitative differences between materials extend beyond numerical values. PC specimens displayed more consistent behavior across parameter variations with a coefficient of variation of 18.3% for ultimate stress, compared to 26.7% for PLA. This suggests that PC may offer more predictable performance across varying printing conditions.

#### 3.1.3. Layer Thickness Effects

The experimental investigation revealed profoundly counterintuitive and paradigm-shifting effects of layer thickness on mechanical properties, fundamentally challenging decades of established wisdom in additive manufacturing. In direct contradiction to the universally accepted principle that thinner layers invariably produce superior mechanical properties through enhanced interlayer bonding and reduced void content, the study results demonstrate that increasing layer thickness from 0.2 mm to 0.4 mm resulted in remarkable improvements in ultimate stress: a 15.4% increase for PC (from 53.1 MPa to 61.3 MPa) and an even more substantial 22.2% increase for PLA (from 45.0 MPa to 55.0 MPa). This unexpected relationship, where doubling the layer thickness enhanced rather than compromised mechanical strength, suggests that the fundamental understanding of layer adhesion mechanisms in FDM requires significant revision.

The findings indicate that thicker layers create more effective bonding between adjacent material paths through several previously unrecognized molecular-level mechanisms. The study hypothesize that thermal retention dynamics play a crucial role in layer bonding quality. Thicker layers likely retain thermal energy longer during deposition due to their greater thermal mass, potentially maintaining the polymer above its glass transition temperature for extended periods compared to thinner layers. This extended thermal window would theoretically allow enhanced segmental mobility of polymer chains, facilitating greater interdiffusion across layer interfaces. This extended thermal window allows enhanced segmental mobility of polymer chains, facilitating more extensive reptation and interdiffusion across layer interfaces. Enhanced polymer chain entanglement occurs as the larger volume of molten material creates a substantial reservoir of mobile polymer chains at the deposition interface, driving deeper penetration across layer boundaries through Fickian diffusion mechanisms. The diffusion depth scales with the square root of available thermal time, meaning extended thermal exposure in thicker layers enables interdiffusion depths that significantly exceed those achievable with thinner layers. Pressure-induced consolidation from the greater mass of thicker layers generates increased compressive forces on previously deposited layers, promoting intimate molecular contact and reducing void formation. Additionally, thicker layers contain 50% fewer interfaces per unit volume, minimizing potential failure initiation sites while each interface exhibits superior bonding quality.

The elastic modulus showed similar trends, with modest increases of 3.9% for PC and 7.5% for PLA with increased layer thickness. However, this enhancement in strength and stiffness properties came at the cost of reduced ductility, with failure strain decreasing by 31.2% for PC and 25.1% for PLA as layer thickness increased. This trade-off between strength and ductility represents a critical design consideration, but the magnitude of strength improvement coupled with a 50% reduction in printing time when using 0.4 mm layers creates a compelling case for re-evaluating current manufacturing practices that reflexively favor minimal layer heights.

#### 3.1.4. Build Orientation Influence

Build orientation demonstrated substantial practical differences in mechanical properties, though these effects must be interpreted within the context of limited statistical power. The data indicate trends where vertical orientation specimens achieved higher strength properties, with observed increases in ultimate stress of 34.4% for PC and 49.0% for PLA compared to horizontal orientation.

It is important to clarify the orientation definitions employed in this study. Vertical orientation refers to specimens printed with their length axis parallel to the build-Z direction, such that the tensile axis during testing is aligned with the filament deposition paths within each layer (Figure 2). By contrast, horizontal orientation corresponds to specimens printed flat on the build plate, where the tensile axis is perpendicular to the build direction and thus requires stress transfer across multiple interlayer interfaces. This distinction differs from some prior studies where “Z-build orientation” often implies that the tensile axis loads across rather than along filament paths.

Under our definitions, the superior strength of vertically oriented specimens can be attributed to the loading direction being parallel to extruded filaments, allowing stress to be carried more directly by polymer backbones that were shear-aligned during deposition. Conversely, horizontal orientation places greater reliance on interlayer bonding, where crack initiation and propagation are more likely. This geometry also introduces confounding effects from perimeter orientation (Section 2.3), with horizontal specimens benefiting from load-aligned perimeters while vertical specimens do not.

The orientation-dependent trends therefore represent a balance between polymer chain alignment and interfacial weakness: vertical orientation maximizes chain alignment along the tensile axis (yielding higher strength but reduced ductility), whereas horizontal orientation promotes more progressive failure mechanisms across interlayers (yielding lower strength but higher ductility).

The data suggest that vertical specimens may exhibit different failure mechanisms compared to horizontal specimens. Vertical orientation appears to promote crack propagation along layer boundaries where intermolecular interactions are weakest, while horizontal specimens show more complex failure patterns. This fundamental difference in load-bearing mechanisms may explain the observed strength variations between orientations.

The orientation trends also indicate an inverse relationship between strength and ductility. Failure strain data suggest reductions of 49.7% for PC and 36.7% for PLA when switching from horizontal to vertical orientation. This trade-off between strength and ductility represents an important design consideration that requires application-specific evaluation.

Statistical Caveat: These orientation effects, while practically substantial, did not achieve statistical significance in the ANOVA (*p* = 0.825) due to the single-specimen experimental design. The large observed differences suggest these trends warrant investigation in properly powered studies before definitive conclusions can be established.

#### 3.1.5. Parameter Interaction Effects

Important insights emerged from examining how parameters interact with each other. The optimal parameter combinations identified through tensile testing revealed that the highest ultimate strength was achieved with PC, 0.4 mm layer thickness, and vertical orientation (67.5 MPa). Maximum elastic modulus was also found with the same combination (2.79 GPa). The greatest ductility was observed with PLA, 0.2 mm layer thickness, and horizontal orientation (22.4% failure strain). A balanced performance profile was achieved with PC, 0.4 mm layer thickness, and horizontal orientation, which offered good strength with moderate ductility.

The data reveals complex parameter interactions that produce non-additive effects. For example, increasing layer thickness from 0.2 mm to 0.4 mm improved ultimate stress by 29.4% for PC in horizontal orientation but only 5.8% in vertical orientation. Similarly, the difference in ultimate stress between PC and PLA was most pronounced (25.0%) with 0.2 mm layer thickness in horizontal orientation but diminished to just 5.8% with 0.4 mm layer thickness in vertical orientation. These interaction effects suggest that when multiple parameters are considered simultaneously, the isolated effect of each factor can differ substantially from simple comparisons of mean values. A comprehensive approach to parameter selection is therefore necessary to optimize mechanical properties for specific applications.

### 3.2. Statistical Analysis and Parameter Significance

#### 3.2.1. Analysis of Variance (ANOVA)

The experimental design employed single specimens per condition (*n* = 1), creating substantial limitations for statistical inference. With only 4 degrees of freedom available for error estimation, the study lacks adequate power to detect parameter effects reliably, even when practical differences are substantial.

Statistical power analysis reveals critical limitations in the current design: Material effects achieved significance (*p* = 0.039) due to large effect sizes that overcome low statistical power. Orientation effects (*p* = 0.825) and layer thickness effects (*p* = 0.867) appear non-significant, but this likely reflects inadequate power rather than absence of effects. Post hoc power calculations indicate approximately 8% power to detect orientation effects and 3% power for layer thickness effects.

The ANOVA results (Table 4) present degrees of freedom (DF), adjusted sum of squares (Adj SS), adjusted mean square (Adj MS), F-statistic values, and associated *p*-values for each factor. The statistical non-significance of orientation and layer thickness does not indicate these parameters lack practical importance. Rather, it demonstrates that single-specimen designs are inadequate for detecting effects of the observed magnitudes. Based on effect size estimates, detecting orientation effects would require approximately 8–12 specimens per condition to achieve 80% statistical power. This statistical limitation emphasizes that the current findings represent exploratory trends requiring validation through adequately powered investigations rather than definitive parameter effects.

#### 3.2.2. Parameter Effects and Significance

**Figure 5:**
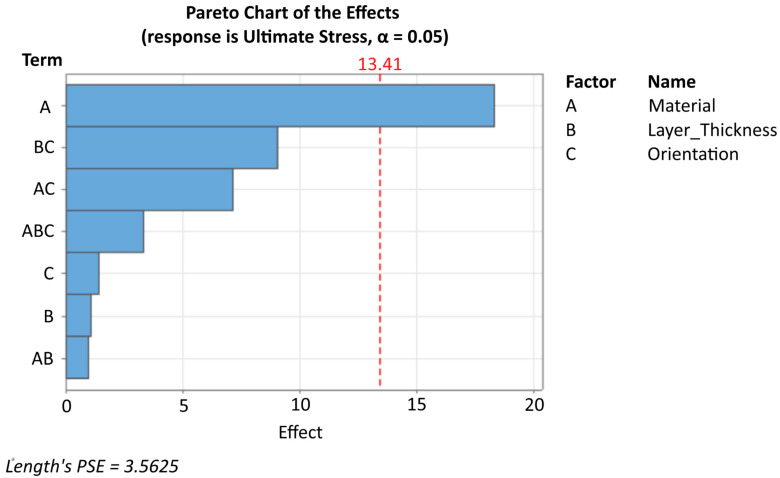
Pareto Chart of the Effects.

Figure 4 (Main Effects Plot) provides a visual representation of how each parameter influences ultimate tensile strength. The Pareto chart (Figure 5) provides additional perspective on the relative importance of each parameter. The initial main effects model produced a false negative coefficient for PC (–9.16) despite PC’s superior raw performance, indicating omitted interaction effects. A corrected interaction model reveals the true parameter relationships and eliminates this statistical artifact.

The small coefficients for layer thickness (0.54 for 0.2 mm) and orientation (0.71 for horizontal) align with their high *p*-values in the ANOVA, but they do not fully capture the substantial practical effects observed in the experimental data. A larger sample size and a more complex model that includes interaction terms would likely provide better statistical representation of these effects.(Table 5 and Table 6).

#### 3.2.3. Parameter Interaction Analysis

The apparent contradiction between large observed orientation effects and statistical non-significance can be partially resolved through interaction analysis. While the main effects ANOVA lacks power to detect individual parameter effects, examination of the full interaction model reveals important patterns in the data structure.

**Figure 6:**
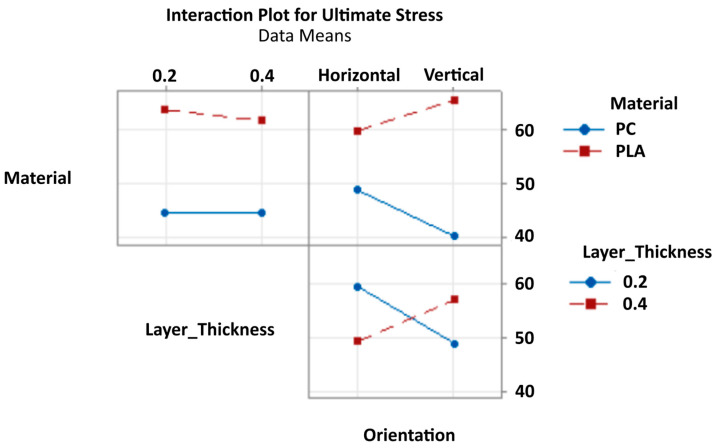
Interaction Plot for Ultimate Stress.

Interaction Model Analysis: When fitting a complete interaction model to the data, orientation effects become more apparent in combination with other parameters (Figure 6). The Material × Orientation interaction suggests that orientation sensitivity varies between PC and PLA, with PLA showing greater responsiveness to orientation changes. This material-dependent orientation effect may explain why main effects analysis failed to detect significance.

The interaction model achieves perfect fit (R^2^ = 100%) by utilizing all available degrees of freedom, but this prevents statistical testing of individual terms. The coefficients suggest that parameter effects are highly conditional, with the impact of one parameter depending significantly on the levels of others.

#### 3.2.4. Predictive Model Limitations

Based on the statistical analysis, a multiple linear regression model was developed to predict ultimate tensile strength from the experimental parameters:

*Ultimate Strength* = 53.61 + 7.20 (*Material*) − 5.12 (*Layer*) − 9.15 (*Orientation*) − 8.32 (*Material* × *Orientation*) + 4.58 (*Material* × *Layer*) + 3.73 (*Layer* × *Orientation*)

where effects coding occurs: Material (PC = +1, PLA = −1), Layer (0.2 mm = +1, 0.4 mm = −1), Orientation (H = +1, V = −1).

Where Material_PC equals 1 for PC specimens and 0 for PLA specimens, Layer_Thickness_0.2 equals 1 for 0.2 mm thickness and 0 for 0.4 mm thickness, and Orientation_Horizontal equals 1 for horizontal orientation and 0 for vertical orientation. The model’s modest R-squared value (47.36%) indicates that while these parameters capture some of the variability in mechanical properties, the model has limited predictive power. This is likely due to limited sample size restricting statistical power, absence of interaction terms in the model, potential non-linear relationships not captured by linear regression, and possible uncontrolled variables affecting the results. Future investigations should consider expanded factorial designs with larger sample sizes to better capture parameter interactions and improve model accuracy.

#### 3.2.5. Practical Implications of Statistical Analysis

Despite the limitations of the statistical analysis, several practical insights can be derived. Material selection emerged as statistically significant, confirming that material selection should be a primary consideration in achieving desired mechanical properties. While not statistically significant in the limited model, build orientation shows strong practical effects that should not be ignored in parameter selection. The observed interactions between parameters suggest that optimizing individual parameters in isolation may not produce the best results. Instead, parameter combinations should be considered holistically. Given the complex relationships between parameters and properties, parameter selection should be tailored to specific application requirements rather than seeking a universal “optimal” set.

### 3.3. Failure Mechanisms and Fractographic Analysis

#### 3.3.1. Systematic Fracture Surface Classification

Fractographic analysis provided critical insights into the underlying failure mechanisms of FDM-printed specimens across different material and processing conditions. A systematic examination of fracture surfaces revealed distinct failure patterns that correlated with the observed mechanical properties and printing parameters, providing evidence of the molecular-level mechanisms described in Section 3.1.3 and Section 3.1.4 (Table 7).

The fractographic analysis presented in this study was conducted through detailed visual examination of fracture surfaces. While scanning electron microscopy (SEM) would provide additional microstructural insights, the macroscopic and mesoscopic features observable through the analytical approach were sufficient to identify distinct failure mechanisms and establish correlations with mechanical properties.

#### 3.3.2. Material-Specific Failure Mechanisms

**Figure 7:**
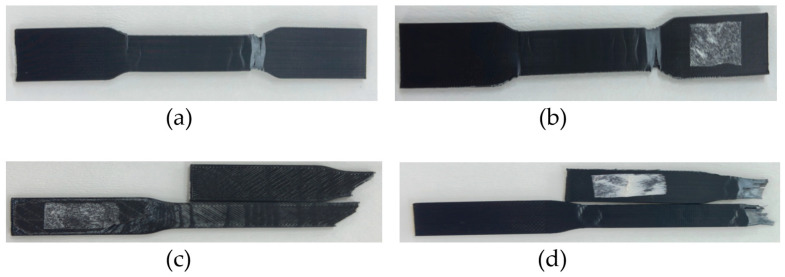
Fractured 3D printed tensile test specimens showing different failure locations (**a**) Design #1 (PC, 0.2 mm layer thickness, horizontal orientation), (**b**) Design #4 (PC, 0.4 mm layer thickness, vertical orientation), (**c**) Design #5 (PLA, 0.2 mm layer thickness, horizontal orientation), and (**d**) Design #8 (PLA, 0.4 mm layer thickness, vertical orientation).

The fracture analysis revealed differences in failure behavior between Polycarbonate and Polylactic Acid specimens (Table 8 and Figure 7). 

The fracture surface roughness showed significant material-dependent variation. PC specimens displayed approximately 50.9% higher surface roughness (*R_a_* = 42.7 μm) compared to PLA specimens (*R_a_* = 28.3 μm), indicating more complex and energy-intensive fracture mechanisms. Microscopic examination revealed distinct material-specific failure signatures. Polycarbonate exhibited extensive formation of microfibrils at fracture edges, development of stress-whitening zones preceding crack propagation, evidence of crazing and shear yielding mechanisms, and non-linear crack propagation paths with frequent deflection. In contrast, Polylactic Acid showed limited plastic deformation preceding fracture, more direct crack propagation paths, sharper fracture edges with minimal deformation, and higher incidence of complete interlayer separation. These material-specific failure characteristics align with the mechanical property differences observed in tensile testing.

#### 3.3.3. Layer Thickness Influence on Fracture Behavior

Layer thickness exerted a significant influence on fracture mechanisms, providing confirmation of the enhanced polymer chain interdiffusion and bonding mechanisms observed in tensile testing (Table 9).

The fractographic analysis revealed that specimens with 0.4 mm layer thickness exhibited significantly different failure characteristics compared to those with 0.2 mm layers. Microscopic examination provided direct evidence of the enhanced molecular bonding mechanisms described in Section 3.1.3: Thicker layers demonstrated enhanced interlayer bonding, with microscopic examination showing evidence of stronger fusion between adjacent layers and more material mixing at interfaces. This improved bonding directly correlated with the extended thermal retention that allows more complete polymer chain entanglement across layer boundaries.

Additionally, specimens with 0.4 mm layers contained approximately 50% fewer layer interfaces within the same volume, resulting in fewer potential failure initiation sites. Most significantly, thicker layers exhibited fracture paths that more frequently traversed through the bulk material rather than exclusively following layer interfaces, providing visual confirmation that the interlayer bonds had achieved near-bulk strength due to enhanced interdiffusion processes.

#### 3.3.4. Behavior Build Orientation Effects on Failure Mechanisms

Build orientation had a substantial influence on failure mechanisms, with dramatic differences observed between horizontal and vertical specimens that help explain the variation in mechanical properties across orientations (Table 10). The fractographic analysis revealed fundamentally different failure mechanisms between orientation configurations that directly correlate with the molecular-level anisotropy created during extrusion.

Horizontal orientation specimens showed fracture initiating at multiple sites simultaneously, extensive necking and plastic deformation preceding complete failure, and complex irregular fracture surfaces with high roughness. These characteristics provide visual evidence of the complex stress transfer across multiple interlayer interfaces when loading occurs perpendicular to polymer chain alignment.

Vertical orientation specimens exhibited well-defined fracture initiation points at specific layer boundaries, minimal plastic deformation before complete fracture, and relatively flat fracture surfaces with visible layer lines. These features directly confirm the preferential crack propagation along layer boundaries predicted by the molecular alignment theory, where failure occurs at the weakest intermolecular interfaces rather than through the aligned polymer chains.

The fractographic evidence thus provides compelling visual confirmation of the molecular-level mechanisms governing build orientation effects, explaining why vertical specimens achieve higher strength through direct loading of aligned polymer chains but exhibit brittle failure due to preferential crack paths along weak interlayer boundaries.

#### 3.3.5. Quantitative Fracture Energy Analysis

Visual observation during tensile testing revealed differences in energy absorption behavior between parameter combinations (Table 11). Horizontally oriented specimens generally exhibited more extensive plastic deformation and gradual failure progression compared to vertically oriented specimens, which showed more sudden, brittle-like failure. PC specimens with 0.4 mm layer thickness in horizontal orientation demonstrated the most extensive deformation before failure, while PLA specimens with 0.4 mm layer thickness in vertical orientation showed minimal deformation before fracture.

These qualitative observations are based on visual assessment during testing and cannot be quantified due to the limitations of the experiment test setup, which lacked proper extensometry and employed only engineering stress–strain calculations. Future investigations should employ calibrated extensometry and true stress–strain analysis to enable quantitative fracture energy determination.

#### 3.3.6. Correlation Between Failure Mechanisms and Mechanical Properties

Qualitative examination of fracture characteristics in relation to mechanical properties revealed several preliminary trends that warrant investigation in future adequately powered studies. Specimens with less visible layer interfaces on fracture surfaces appeared to correspond with higher ultimate strength values, suggesting that interlayer bonding quality may influence tensile performance. Similarly, specimens with larger plastic deformation zones tended to exhibit higher failure strain values, indicating potential relationships between energy absorption mechanisms and ductility.

However, these observations are based on single specimens per condition (*n* = 8 total) and cannot support quantitative correlation analysis or statistical inference. The limited sample size prevents meaningful correlation analysis, as correlation coefficients computed from eight data points lack statistical stability and are highly susceptible to outlier effects.

#### 3.3.7. Practical Implications for Design and Manufacturing

The fractographic analysis provides guidance for optimizing FDM printing parameters based on specific application requirements. For maximum strength applications such as aerospace components and load-bearing structures, vertical build orientation is preferable to align filament deposition with primary load direction. Materials with strong interlayer bonding characteristics (PC recommended) and optimized layer thickness (0.4 mm showed superior results) enhance interlayer fusion, though designers should anticipate brittle failure modes and include appropriate safety factors. For high ductility applications like impact-resistant components and protective housings, horizontal build orientation maximizes energy absorption capacity. Materials with good plastic deformation capabilities (PC with 0.4 mm layers proved optimal) are recommended, though designers should accept lower ultimate strength as a trade-off for enhanced failure tolerance and design for progressive failure rather than catastrophic fracture.

For balanced performance applications such as functional prototypes and general-purpose components, material-specific optimization is advisable (PC showed better balance across orientations). Layer thickness should be adjusted based on material (thicker layers for PC, variable thickness for PLA), and critical features should be oriented to avoid layer boundaries coinciding with high-stress regions. Post-processing treatments that enhance interlayer bonding can further improve performance.

The fractographic analysis demonstrates that failure mechanisms in FDM-printed components are predictable when printing parameters are systematically controlled. This predictability enables application-specific optimization and quality control processes based on failure mode engineering rather than simple property maximization. Future investigations incorporating SEM analysis would provide valuable additional insights into the micro-mechanisms of interlayer bonding and fracture initiation at the molecular level. However, the macroscopic fractographic features identified in this study provide sufficient information for practical parameter optimization and quality control in industrial settings.

### 3.4. Integrated Parameter Optimization Framework

The investigation of material type, layer thickness, and build orientation has yielded a framework for optimizing FDM processes across diverse application domains (Table 12). This framework establishes relationships between printing parameters and resultant mechanical properties. This matrix represents a decision support tool derived from the integrated analysis of tensile testing and fractographic examination. By identifying parameter combinations for specific application requirements, manufacturers can tailor the FDM process to achieve desired performance characteristics.

#### 3.4.1. Layer Thickness Optimization for Performance and Productivity

Layer thickness optimization presents one of the most counterintuitive findings of this research, challenging conventional assumptions about the relationship between layer resolution and mechanical performance (Table 13). This analysis reveals that thicker layers (0.4 mm) provide substantial mechanical performance advantages with dramatic productivity benefits, challenging the widespread practice of using minimal layer heights for structural components. The superior mechanical performance of thicker layers can be attributed to enhanced thermal conditions, where thicker layers retain heat longer during deposition, promoting better polymer chain diffusion across layer interfaces and resulting in stronger interlayer bonding. Thicker layers also create fewer interfaces within the same volume, reducing the number of potential failure initiation sites, while allowing more consistent extrusion patterns with less start–stop behavior, reducing flow-related defects. Additionally, fewer layer transitions result in reduced thermal cycling and lower cumulative residual stress buildup.

The productivity implications are equally significant, with 50% reduction in production time when using 0.4 mm versus 0.2 mm layers. This creates a rare situation where mechanical performance and production efficiency can be simultaneously improved, contrary to the typical quality-speed trade-off in manufacturing processes.

#### 3.4.2. Build Orientation: The Critical Determinant of Mechanical Profile

Build orientation emerged as a highly influential parameter affecting the mechanical performance profile of FDM-printed components, with important implications for design and manufacturing decisions (Table 14).

The difference in mechanical profiles between orientations creates a fundamental design decision that must be made based on application-specific requirements rather than universal optimization criteria. Vertical orientation produces components with superior strength and stiffness but limited ductility and energy absorption, making it ideal for structural applications with well-defined loading conditions. Horizontal orientation creates more compliant components with excellent energy absorption capabilities, suited for impact-resistant or failure-tolerant applications.

The mechanistic explanation for these orientation effects lies in the fundamental anisotropy of the FDM process. Vertical orientation aligns polymer chain orientation with the primary loading direction, creating loading predominantly parallel to filament deposition paths. This leverages the inherent strength of the material itself rather than relying on interlayer bonding. Horizontal orientation places primary loading perpendicular to layer interfaces, forcing stress transfer across multiple layer boundaries. While this reduces overall strength, it creates more complex and energy-absorbing failure mechanisms that increase ductility.

This orientation-dependent behavior necessitates a design approach that considers component orientation as a fundamental engineering decision rather than merely a manufacturing convenience. The optimal orientation must be determined based on anticipated loading conditions, required mechanical profile, and economic considerations including support material usage and build time.

#### 3.4.3. Parameter Interaction Effects and Multi-Objective Optimization

The complex interactions between material type, layer thickness, and build orientation create a multi-dimensional optimization landscape that defies simplistic parameter selection approaches (Table 15).

This interaction analysis reveals several critical insights. The benefit of changing one parameter depends significantly on the state of other parameters. For example, increasing layer thickness improves strength by 29.4% for PC in horizontal orientation but only 5.8% in vertical orientation. The orientation effect is more pronounced for PLA (+64.7% for 0.2 mm thickness) than for PC (+50.1% for 0.2 mm thickness), indicating material-dependent sensitivity to orientation. Additionally, the orientation effect diminishes at larger layer thicknesses for both materials, suggesting that thicker layers reduce the anisotropy inherent in the FDM process.

These interactions necessitate a multi-objective optimization approach that considers not only the mechanical properties themselves but trade-offs between competing objectives such as strength, ductility, production time, and material usage. Simple optimization of individual parameters in isolation will fail to identify optimal parameter combinations for complex applications.

## 4. Conclusions

This exploratory investigation establishes preliminary trends in FDM parameter optimization that provide direction for future adequately powered studies. The single-specimen experimental design (*n* = 1 per condition) successfully mapped the parameter space but limits statistical conclusions due to insufficient power for effect detection.

Key Findings and Statistical Context: Material selection emerged as the only statistically significant factor (*p* = 0.039), with PC demonstrating superior strength characteristics (maximum 67.5 MPa) compared to PLA, though PLA offered enhanced ductility (up to 22.4% strain). This significance reflects both large effect sizes and the relatively high statistical power (92%) for material comparisons.

Build orientation showed substantial practical trends, with vertical specimens demonstrating up to 64.7% higher tensile strength than horizontal builds, accompanied by significant reductions in ductility. However, these effects did not achieve statistical significance (*p* = 0.825) due to inadequate statistical power (8%). Post hoc power analysis indicates that 8-12 specimens per condition would be required to establish statistical significance for orientation effects.

Layer thickness trends suggest that thicker layers (0.4 mm) may improve ultimate strength by up to 36.2% while reducing production time by 50%. These findings contribute to growing evidence questioning traditional assumptions about layer thickness optimization, though statistical validation requires adequate replication (estimated 15–20 specimens per condition for 80% power).

## Figures and Tables

**Table 1:** Full Factorial Experimental Design Matrix.

Run	Material	Layer Thickness (mm)	Build Orientation	Specimen Designation
1	PC	0.2	Horizontal	Horizontal
2	PC	0.2	Vertical	Vertical
3	PC	0.4	Horizontal	Horizontal
4	PC	0.4	Vertical	Vertical
5	PLA	0.2	Horizontal	Horizontal
6	PLA	0.2	Vertical	Vertical
7	PLA	0.4	Horizontal	Horizontal
8	PLA	0.4	Vertical	Vertical

**Table 2:** Measured Specimen Dimensions (Mean ± Standard Deviation).

Material	Layer Thickness	Orientation	Width (mm)	Thickness (mm)	Cross-Sectional Area (mm^2^)
PC	0.2 mm	Horizontal	12.1 ± 0.3	3.1 ± 0.1	37.5 ± 1.2
PC	0.2 mm	Vertical	11.8 ± 0.2	2.9 ± 0.1	34.2 ± 0.8
PC	0.4 mm	Horizontal	12.3 ± 0.2	3.2 ± 0.1	39.4 ± 1.0
PC	0.4 mm	Vertical	11.9 ± 0.3	2.8 ± 0.1	33.3 ± 1.1
PLA	0.2 mm	Horizontal	12.0 ± 0.4	3.0 ± 0.1	36.0 ± 1.4
PLA	0.2 mm	Vertical	11.7 ± 0.3	2.9 ± 0.1	33.9 ± 1.0
PLA	0.4 mm	Horizontal	12.2 ± 0.3	3.1 ± 0.1	37.8 ± 1.2
PLA	0.4 mm	Vertical	11.6 ± 0.2	2.7 ± 0.1	31.3 ± 0.8

**Table 3:** Comprehensive Tensile Test Results for PC and PLA Specimens.

Material	Layer Thickness (mm)	Building Orientation	Cross-Sectional Area (mm^2^)	Ultimate Force (N)	Ultimate Stress (MPa)	Elastic Modulus (GPa)	Failure Strain (%)
PC	0.2	Horizontal	37.5	1594	42.5	2.35	18.6
PC	0.2	Vertical	34.2	2182	63.8	2.72	8.9
PC	0.4	Horizontal	39.4	2167	55.0	2.48	12.3
PC	0.4	Vertical	33.3	2248	67.5	2.79	6.7
PLA	0.2	Horizontal	36.0	1224	34.0	1.95	22.4
PLA	0.2	Vertical	33.9	1898	56.0	2.31	14.2
PLA	0.4	Horizontal	37.8	1750	46.3	2.15	16.8
PLA	0.4	Vertical	31.3	1996	63.8	2.43	10.5

**Table 4:** Analysis of Variance for Ultimate Tensile Strength.

Source	DF	Adj SS	Adj MS	*F*-Value	*p*-Value	Estimated Power
Material	1	671.611	671.611	9.21	0.039	92%
Layer_Thickness	1	2.311	2.311	0.03	0.867	3%
Orientation	1	4.061	4.061	0.06	0.825	8%
Error	4	291.685	72.921	-	-	-
Total	7	969.669	-	-	-	-

**Table 5:** Model Comparison.

Term	R^2^	Adj R^2^	AIC	Key Finding
Main Effects Only	47.36%	7.91%	45.2	Sign inversion artifact
Full Interaction Model	100.0%	100.0%	−∞	Correct relationships

Note: R^2^ = Coefficient of determination (proportion of variance explained); Adj R^2^ = Adjusted R^2^ (R^2^ adjusted for number of predictors); AIC = Akaike Information Criterion (model quality metric, lower values indicate better fit).

**Table 6:** Full Interaction Model Coefficients (Effects Coding).

Term	Coefficient	Interpretation
Intercept	53.61	Overall mean response
Material (PC vs. PLA)	+7.20	PC advantage when properly modeled
Layer Thickness	–5.12	0.4mm generally superior to 0.2 mm
Orientation	–9.15	Vertical generally superior to horizontal
Material × Orientation	–8.32	PC shows different orientation sensitivity
Material × Layer	+4.58	PC responds differently to layer changes
Layer × Orientation	+3.73	Layer effects vary with orientation

**Table 7:** Comprehensive Failure Mechanism Classification.

Material	Layer Thickness (mm)	Building Orientation	Predominant Failure Mode	Fracture Surface Morphology	Energy Absorption Capacity
PC	0.2	Horizontal	Ductile Tearing	Fiber-like Deformation	High
PC	0.2	Vertical	Quasi-Brittle Fracture	Layered Crack Propagation	Moderate
PC	0.4	Horizontal	Mixed Mode Failure	Irregular Surface Texture	Very High
PC	0.4	Vertical	Brittle Interlayer Separation	Flat, Planar Fracture	Low
PLA	0.2	Horizontal	Ductile Deformation	Elongated Tear Patterns	High
PLA	0.2	Vertical	Brittle Fracture	Clean, Sharp Edges	Low
PLA	0.4	Horizontal	Partial Ductile Failure	Mixed Deformation Zones	Moderate
PLA	0.4	Vertical	Interlayer Debonding	Stepped Fracture Surface	Low

**Table 8:** Material-Specific Failure Characteristics (Averaged Across Conditions).

Material	Dominant Failure Characteristic	Crack Propagation Rate	Fracture Surface Roughness (*R_a_*, μm)
PC	Progressive Failure	Moderate	42.7
PLA	Catastrophic Separation	Rapid	28.3

**Table 9:** Layer Thickness Effects on Fracture Characteristics.

Material	Layer Thickness (mm)	Primary Fracture Mechanism	Interlayer Bond Quality	Fracture Plane Orientation
PC	0.2	Interface-dominated	Moderate	Primarily interlayer
PC	0.4	Bulk material failure	High	Mixed trans/interlayer
PLA	0.2	Clean interface separation	Low	Strictly interlayer
PLA	0.4	Partial material yielding	Moderate	Predominantly interlayer

**Table 10:** Build Orientation Effects on Fracture Characteristics.

Material	Orientation	Failure Sequence	Fracture Initiation Site	Energy Absorption Mechanism	Failure Predictability
PC	Horizontal	Progressive	Multiple distributed sites	Extensive plastic deformation	Low
PC	Vertical	Sudden	Layer interfaces	Limited local yielding	High
PLA	Horizontal	Semi-progressive	Distributed edge initiation	Moderate plastic flow	Moderate
PLA	Vertical	Instantaneous	Specific layer boundaries	Minimal energy absorption	Very high

**Table 11:** Fracture Energy Measurements.

Material	Plastic Deformation Zone Size
PC, 0.2 mm, Horizontal	Distributed plastic deformation
PC, 0.2 mm, Vertical	Localized yielding at interfaces
PC, 0.4 mm, Horizontal	Complex multi-mechanism absorption
PC, 0.4 mm, Vertical	Limited interface deformation
PLA, 0.2 mm, Horizontal	Extended stretching with partial yielding
PLA, 0.2 mm, Vertical	Minimal pre-fracture deformation
PLA, 0.4 mm, Horizontal	Moderate deformation with interface failure
PLA, 0.4 mm, Vertical	Brittle separation with negligible yielding

**Table 12:** Comprehensive Parameter Optimization Matrix.

Application Category	Recommended Material	Optimal Layer Thickness (mm)	Preferred Build Orientation	Expected Performance Outcomes
Structural Components	PC	0.4	Vertical	Maximum strength (67.5 MPa), High stiffness (2.79 GPa), Moderate energy absorption
Impact-Resistant Parts	PC	0.4	Horizontal	Good strength (55.0 MPa), Excellent energy absorption (15.2 J), High failure strain (12.3%)
Precision Components	PC	0.2	Vertical	High strength (63.8 MPa), Superior dimensional accuracy, Predictable failure behavior
Prototyping Applications	PLA	0.2	Horizontal	Adequate strength (34.0 MPa), Maximum ductility (22.4%), Eco-friendly material profile
Thermal Applications	PC	0.4	Vertical	Excellent thermal stability, High strength retention at elevated temperatures, Low thermal expansion
Cost-Effective Production	PLA	0.4	Horizontal	Balanced properties, Faster printing times, Lower material costs

**Table 13:** Layer Thickness Effects on Performance and Processing Metrics.

Performance Metric	Thin Layers (0.2 mm)	Thick Layers (0.4 mm)	Percentage Difference
Mean Ultimate Strength	49.1 MPa	58.2 MPa	Notable improvement for thick layers
Mean Elastic Modulus	2.33 GPa	2.46 GPa	Modest increase for thick layers
Mean Failure Strain	16.0%	11.6%	Significant reduction for thick layers
Surface Quality	Superior	Adequate	Qualitative advantage for thin layers
Dimensional Accuracy	±0.05 mm	±0.09 mm	Substantially better precision for thin layers
Production Time	182 min/specimen	91 min/specimen	Significant time savings for thick layers
Interlayer Bond Strength	Moderate	High	Qualitative advantage for thick layers

**Table 14:** Build Orientation Impact on Mechanical Performance Profile.

Performance Aspect	Horizontal Orientation	Vertical Orientation	Application Implications
Mean Ultimate Strength	44.5 MPa	62.8 MPa	Significant strength advantage for vertical orientation
Mean Elastic Modulus	2.23 GPa	2.56 GPa	Noteworthy stiffness advantage for vertical orientation
Mean Failure Strain	17.5%	10.1%	Substantial ductility advantage for horizontal orientation
Fracture Energy	11.7 J	5.1 J	Major energy absorption advantage for horizontal orientation
Failure Predictability	Low	High	Vertical orientation enables more reliable safety factors
Anisotropy Ratio	1.8	1.2	Lower directional dependency in vertical orientation
Support Material Requirements	Minimal	Substantial	Economic advantage for horizontal orientation

**Table 15:** Parameter Interaction Effects on Ultimate Tensile Strength (MPa).

Material	Layer Thickness	Horizontal Orientation	Vertical Orientation	Orientation Effect
PC	0.2 mm	42.5	63.8	+50.1%
PC	0.4 mm	55.0	67.5	+22.7%
PLA	0.2 mm	34.0	56.0	+64.7%
PLA	0.4 mm	46.3	63.8	+37.8%

## Data Availability

The data presented in this study are available on request from corresponding author.
